# Mechanism of Optical and Electrical H_2_S Gas Sensing of Pristine and Surface Functionalized ZnO Nanowires

**DOI:** 10.1021/acsomega.4c04412

**Published:** 2024-12-12

**Authors:** Angelika Kaiser, Tanja Mauritz, Joachim Bansmann, Johannes Biskupek, Ulrich Herr, Klaus Thonke

**Affiliations:** † Institute of Functional Nanosystems, University Ulm, Albert-Einstein-Allee 47, 89081 Ulm, Germany; ‡ Semiconductor Physics Group, University Ulm, 89081 Ulm, Germany; § Institute for Surface Science and Catalysis, University Ulm, 89081 Ulm, Germany; ∥ Electron Microscopy Group of Materials Science, University Ulm, 89081 Ulm, Germany

## Abstract

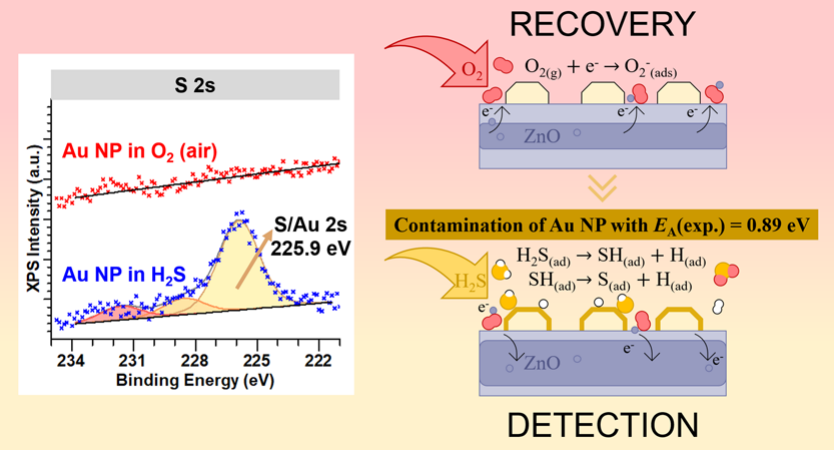

In this work, the sensing ability and the underlying reaction pathways of H_2_S adsorption on two nanomaterial systems, pristine zinc oxide (ZnO) nanowires (NWs) and gold functionalized zinc oxide nanowires (Au@ZnO NWs), were explored in a side-by-side comparison of optical and electrical gas sensing. The properties of optical sensing were analyzed by photoluminescence intensity-over-time measurements (*PL*-*t*) of as-grown ZnO NW samples, and the electrical gas-sensing properties were analyzed by current-over-time measurements (*I*-*t*) of ZnO NW chemically sensitive field-effect transistor (ChemFET) structures with a gas-sensitive open gate. The ZnO NWs were grown by high-temperature chemical vapor deposition (CVD) and thereafter surface-functionalized with a thin Au nanoparticle layer by magnetron sputtering. Detailed X-ray photoelectron spectroscopy (XPS) analysis, alongside an experimental estimation of activation energies (*E*_A_) involved in the H_2_S sensing process, and the application of a simple analytical test allowed us to propose a complete picture of the sensing mechanism on the pristine ZnO surface and the Au@ZnO surface. The combined results hint at H_2_S dissociation via surface interaction and irreversible adsorption dynamics for both material systems occurring already at room temperature. Our findings specifically emphasize the impact of Au functionalization morphology on sensor sensitivity and the beneficial importance of chemical affinity between Au and H_2_S for superior H_2_S sensing results, aiming at enhanced response and selectivity for potential medical H_2_S detection in human breath.

## 1 Introduction

Hydrogen sulfide is a unique biomarker of diseases in the human body.^1^ Healthy exhaled human breath contains nitrogen (N_2_), oxygen (O_2_), carbon dioxide (CO_2_), thousands of volatile organic compounds (VOC), and a prominent sulfide, namely H_2_S, at a low concentration of 0–1.3 ppb.^1^ The ability to detect variations in trace amounts of H_2_S in exhaled human breath with a multiarray sensor device (e.g. “e-nose”^2^) could enable noninvasive early diagnostics and therapeutic monitoring of chronic pulmonary diseases, like asthma.^3^

Cost-efficient sensing and direct analysis of breath samples can be achieved by gas-responsive nanoscale metal oxide semiconductors (MOS), which can be utilized in optical and electrical sensor setups. MOS sensors come with a variety of advantages for research, fabrication, and application: Optical MOS sensors benefit from the overall simplicity of sensor fabrication because the as-grown nanomaterial can be directly utilized, and no additional sample preparation or contact structure is required.^4^ Here, the measured sensing signal is based on the modification of MOS material properties itself because no electronics or optoelectronics are implemented. Meanwhile, electrical MOS sensors and readout circuits are compact and easy to operate. The small size greatly facilitates their incorporation into already existing electrical devices, e.g. medical e-noses^5^ and smartphones,^6^ or their application on fabric.^7^

Among the reported MOS, ZnO is a good candidate for a potent H_2_S sensing material system, based on its common application as an efficient H_2_S sorbent^8^ even at ambient temperature.^9^ The superior H_2_S uptake by ZnO at low temperatures can be enhanced even further by the introduction of doping,^10^ surface functionalization with noble metals,^11^ and improvement of the surface-to-volume ratio via a decrease in crystal size.^12^ Sensors based on functionalized ZnO were reported to detect H_2_S concentrations below the ppb level and at temperatures far below the optimum reaction temperature of H_2_S with ZnO, which is reported to be around 200–400 °C.^13,14^

Despite the vast number of publications in the field of breath analysis with MOS,^15,16^ gas sensing results can still be highly empirical and unpredictable.^1^ Proposed gas sensing mechanisms of pristine MOS or surface functionalized MOS are frequently mere suggestions based on muddled or lacking information about material morphology and adsorption dynamics. Results must often be considered on a case-by-case basis.

In this work, we provide detailed insight into morphology, surface chemistry, sensing ability, and the underlying reaction pathways of H_2_S adsorption of high-temperature CVD-grown ZnO NWs at sensing temperatures below 100 °C. We test the gas sensing performance of pristine ZnO and surface functionalized ZnO in pure N_2_, pure O_2_, and 1 ppm of H_2_S diluted in N_2_. Our findings on the H_2_S sensing mechanism of both material systems are based on a comparative study and analysis of two gas sensing methods: optical and electrical gas sensing in the form of *PL*-*t* measurements of contactless as-grown samples, and *I*-*t* measurements of ChemFET structures with Au contacts. For direct comparison, all sensors are prepared from a single NW growth sample, ensuring the same crystalline quality and morphology throughout all optical and electrical sensing experiments. We perform gas sensing measurements at different sensor temperatures to derive the underlying apparent *E*_A_s, which are determined by the slowest processes in the H_2_S sensing mechanism. The measured *E*_A_s hint to a dissociative chemisorption of H_2_S on both surface types.

We find that the sensitivity of our pristine ZnO NW and Au@ZnO NW sensors improves with increasing temperature, suggesting that the H_2_S adsorption dynamics must follow an irreversible process on both surfaces. This is derived from an analytical test, introduced by Lee et al.,^17^ which, when applied to our results, indicates an irreversible component in the sensing mechanism of the ZnO NW system and explains their sub-ppb sensing ability. The irreversible nature of H_2_S sensing via Au@ZnO surface has already been suggested by our previous results.^18,19^ The measurements are now complemented with transmission electron microscopy (TEM) and XPS data.

The estimated *E*_A_s, the irreversible adsorption characteristics, a clear XPS analysis of the Au@ZnO NW surface reactivity to H_2_S exposure, and last the side-by-side comparison of optical and electrical H_2_S sensing results allow us to better understand the origin of the superior sensitivity and selectivity of Au functionalized ZnO. Based on these results, we propose a complete picture of the sensing mechanism of pristine ZnO NWs and Au@ZnO NWs toward H_2_S.

## 2 Method

### 2.1 ZnO NW Growth

ZnO NWs were grown by high temperature chemical vapor deposition (CVD) using vapor liquid solid (VLS) growth on silicon (100) substrate with a 3 nm Au catalyst layer. The source material was a mixture of ZnO powder (Alfa Aesar, purity: 99.99 %) and graphite powder (Alfa Aesar, purity: 99.9999 %) in a molar ratio of 1:1. The substrate had a dimension of 1 cm^2^, and 300 mg of source material were used for one growth run.

The growth setup was a horizontal tube furnace with three heating zones, each being 20 cm long. After reaching the desired temperature profile, the complete tube furnace was moved along the 2 m long and 4 cm wide quartz glass growth tube. The source material was heated up to 1045 °C and the evaporated zinc (Zn) was carried via argon flux (Ar, 99.998 % purity, 190 sccm flow rate) to the growth substrate, which was placed at a 25 cm distance and kept at 1057 °C. Here, Zn vapor formed a liquid alloy with the Au catalyst layer. An oxygen inlet (O_2_, 99.995 % purity diluted in Ar down to 5%, 1.14 sccm flow rate) at 24 cm from the source material enabled the selective oxidation of the alloy and thus the formation of ZnO NWs. The growth time was set to 60 min at a pressure of 900 mbar.

**Figure 1:**
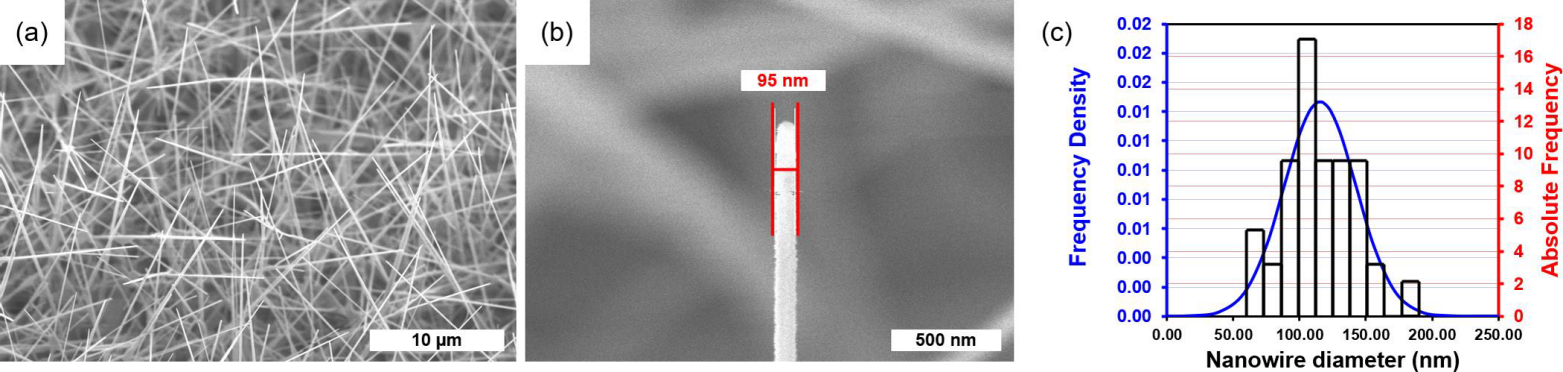
(a,b) Scanning electron images of pristine ZnO NWs grown by high temperature CVD on Si (100) with Au catalyst. (c) ZnO NW diameter distribution with a peak diameter of 110 nm.

The growth resulted in a homogeneous distribution of ZnO NWs, as depicted in scanning electron micrographs in Figure 1. The NWs were about 30–40 μm long and ∼110 nm thick.

### 2.2 Gas Sensor Sample Fabrication

Both, pristine ZnO NWs and Au@ZnO NWs, were tested in a computer-controlled gas sequence at four different temperatures for their optical gas sensing properties, and at three different temperatures in a second setup for their electrical gas response. For better comparability of different measurement conditions, optical and electrical sensor samples were prepared from a single NW growth sample, ensuring the same crystalline quality and morphology of ZnO NWs. Therefore, one growth sample with homogeneous morphology and base PL intensity was selected and cleaved into multiple smaller pieces. These pieces were subsequently assigned to the measurements, i.e. optical and electrical, respectively.

For *optical* gas sensing measurements, at each measurement temperature, a separate sample piece was used (4 in total). We measured each piece once before surface functionalization to determine the sensing ability of pristine ZnO NWs, and a second time after surface functionalization with Au to find the sensing ability of Au@ZnO NWs. Surface Au functionalization of the as-grown pristine ZnO NWs was performed via magnetron sputtering, as mentioned in section 2.3.

*Electrical* gas sensing measurements with pristine and functionalized ZnO were performed by using sensors based on ChemFET structures with open gate. For the pristine ZnO NW sensor fabrication, NWs were scratched off from growth sample pieces and dissolved in isopropanol. The ZnO/isopropanol suspension was then drop-coated onto a planar contact structure, which was formed by two 5 mm long and parallel Ti/Au electrodes separated by a 5 μm gap. These contact structures were fabricated by using conventional optical lithography on glass substrate. The alignment of ZnO NWs across the gap was achieved via dielectrophoresis. Electrical sensors with functionalized Au@ZnO NWs were fabricated by surface functionalization of freshly prepared ZnO NW sensors via magnetron sputtering (section 2.3). As nanowires are drop-coated and aligned on different contact structures, the base current of these sensors can still differ considerably. Based on this consideration, to ensure good comparability throughout all electrical measurements, two sensors were fabricated and investigated. The first sensor was used for sensing measurements of pristine ZnO NWs at all temperatures. The second sensor was Au functionalized and measured at all temperatures as well.

### 2.3 Au Functionalization

The surface functionalization was performed with a Bal-Tec Med 020 high vacuum coating system. The sputtering parameters for room temperature deposition of a 3 nm thick Au nanoparticle layer were 7 × 10^–3^ Pa working pressure in Ar atmosphere, 7 W power, 17 s sputtering time, and a 2” target size (99 % purity Au). The deposition rate was initially calibrated by X-ray reflectometry (XRR) measurements using test samples. For gas sensing samples, a thin Au nanoparticle layer of only 3 nm thickness was chosen. With a thin functionalization layer, the signal intensity loss in room temperature (RT) PL measurements due to the reflective Au functionalization layer is minimized, as well as the risk of creating a short circuit on the electrical gas sensors.^20^

### 2.4 Measurement

In this work, the ambient gases tested in the optical and electrical sensing experiments were pure nitrogen (N_2_, 99.9999 % purity), pure oxygen (O_2_, 99.995 % purity), and 1 ppm of H_2_S (98 % purity) prediluted in nitrogen (N_2_, 99.9999 % purity). Samples are exposed to a predefined gas sequence. The exposure to different gas atmospheres and their alteration is based on dynamic gas flow, meaning that samples are flushed with a continuous and stable gas flow of set concentration and composition at all times. Gas sensing results of gas sensors based on ZnO NWs and Au@ZnO NWs for the detection of ppb-concentrations of H_2_S prediluted in synthetic air, as well as for selectivity studies on ppm-concentrations of CH_4_ and H_2_, were reported in our previous works.^18,19^

The details of our optical and electrical measurement setups and methods are explained in the following sections.

#### 2.4.1 Optical Setup

A detailed depiction of the optical setup is displayed in Figure S1a. Room temperature (RT) PL-based H_2_S sensing of as-grown ZnO NWs and Au@ZnO NWs was performed in a dynamic flow chamber with a volume of 22 ml. The gas flow chamber could be heated via a heating filament, controlled by a Pt 100 sensor and a temperature controller. The measurement chamber was gastight, and a Suprasil glass window allowed direct optical excitation of the samples with a CW solid-state laser, which was operated at a wavelength of 320 nm. *PL*-*t* data were recorded with a Horiba Spectrum ONE CCD camera attached to a Spex 270 M monochromator with a focal length of *f* = 270 mm. The change of the ambient gases greatly affected the intensity of the excitonic near-band-edge (NBE) emission of ZnO around 380 nm, as seen in Figure S2a. Hence, the gas-sensitive PL intensity in the *PL*-*t* measurements was defined as the integrated NBE emission, which also ensured an improved signal-to-noise ratio (Figure S2b). The spectra acquisition rate was set to 0.2/s, and the sensor temperatures were set to 310 K, 320 K, 330 K, and 340 K. In the optical setup, switching between different gas atmospheres was performed manually.

#### 2.4.2 Electrical Setup

A detailed depiction of the electrical setup is displayed in Figure S1b. The electrical gas sensing properties of pristine ZnO NWs and Au@ZnO NWs were measured in a sealed dynamic flow chamber with a total volume of 14 ml. The fast switching between different gas atmospheres for continuous detection cycles was managed by a computer-controlled mixing stage, which consisted of various mass flow controllers and a pressure controller. Heating resistors, which were attached to the bottom of each measurement site, a Pt 100 sensor, and an Oxford ITC4 temperature controller enabled to switch between various working temperatures of the setup. *I*-*t* measurements for each sensor were performed at three different temperatures of 305 K, 315 K, and 325 K. The sensor current at an applied bias voltage of 1 V was measured via self-build computer-switchable low-noise current amplifiers at an acquisition rate of 1/s*.*

### 2.5 Gas Sensor Evaluation

*PL*-*t* data and *I*-*t* data were acquired by sequential exposure of NW growth samples and ChemFET sensors to pure O_2_, N_2_, or 1 ppm of H_2_S in N_2_. The gas sensing performances during optical and electrical measurements were evaluated by setting a reference PL intensity level *PL*_0_ or reference current level *I*_0_, respectively. The gas response *R* was defined as the maximum PL intensity change Δ*PL*:
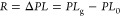
1or the maximum current change Δ*I*:
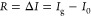
2where *PL*_g_ and *I*_g_ stand for the maximum PL intensity (*PL*_g_) or current (*I*_g_) in the test gas atmosphere. In the case of a sensor with a stable reference level and for easier comparability of the optical and electrical response, the data is usually presented in its normalized form as
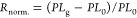
3or:
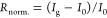
4

## 3 Results

### 3.1 TEM Characterization

To identify specific processes responsible for the gas sensing mechanism, it is crucial to characterize the morphology, crystalline quality, and composition of the material system. In this work, we investigated the H_2_S gas sensing properties of pristine ZnO(10–10) NW surface and of Au nanoparticles supported on ZnO(10–10) NW surface. The ZnO NW and Au surface functionalization were investigated using (scanning) transmission electron microscopy (TEM) together with energy dispersive X-ray (EDX) spectroscopy. The TEM sample preparation was similar to the optical and electrical Au@ZnO NW sensor preparation: First, pristine ZnO NWs were loosely dispersed on a TEM grid (copper mesh) and then covered by an Au nanoparticle layer using magnetron sputtering. TEM investigations were carried out using a Thermofisher Talos 200X microscope operated at 200 kV. The TEM was equipped with a SuperX EDX detector.

**Figure 2:**
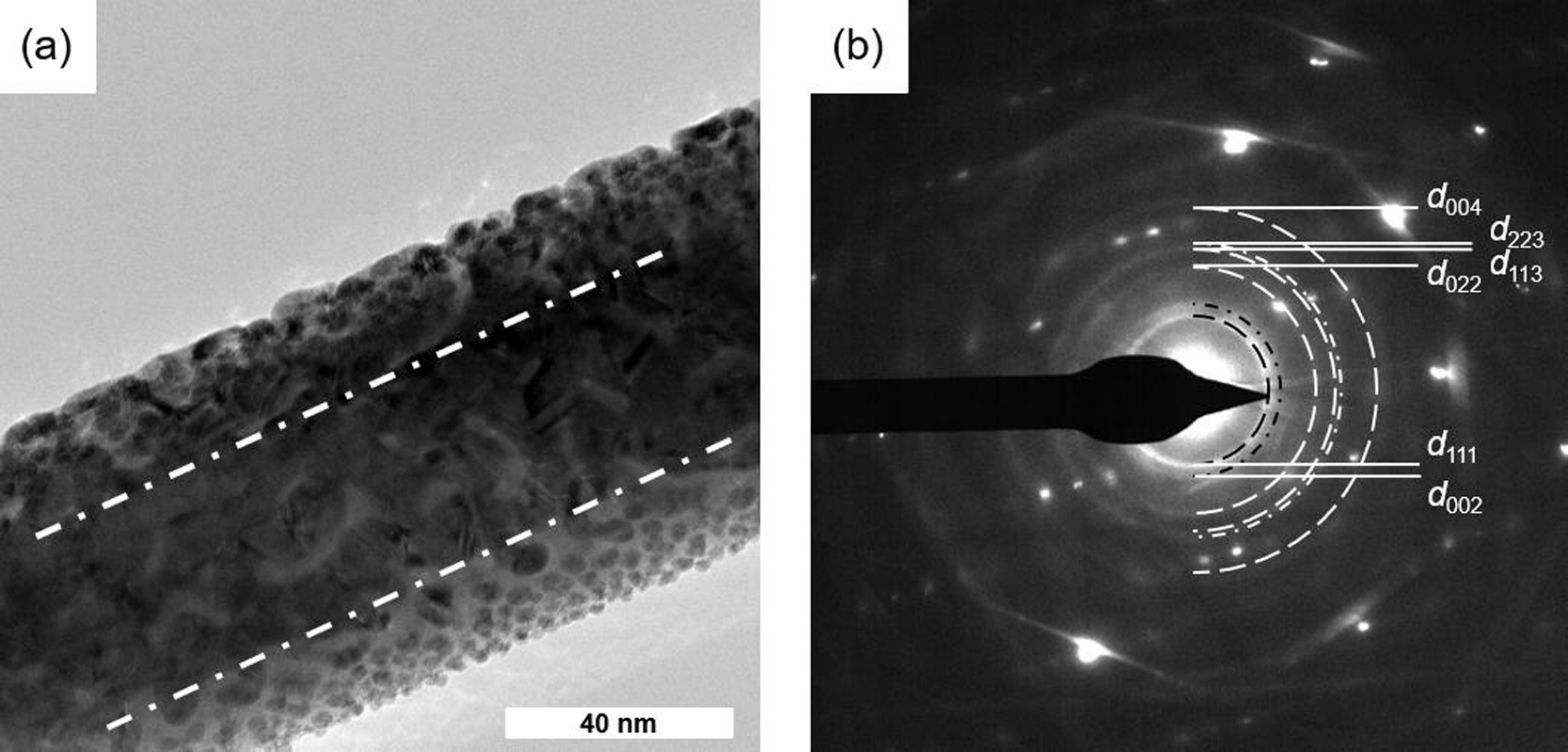
(a) Transmission electron images of a typical Au@ZnO NW with Au functionalization nanoparticles distributed on different m-plane facettes (white broken lines). (b) Corresponding SAED pattern of monocrystalline hcp ZnO NW (spot pattern) and polycrystalline fcc Au functionalization (ring pattern).

Figure 2a shows a bright field TEM image of an Au@ZnO(10–10) NW. For most dispersed ZnO NWs, one m-plane was approximately oriented parallel to the sputter target. As a result, the functionalization layer morphology is orientation-dependent, and differently oriented m-plane facets show different Au nanoparticle arrangements and densities. Whereas the top m-plane facet was covered with a nanoparticle layer consisting of densely packed polycrystalline Au nanoislands, the sideward-oriented m-plane facets show much smaller and less densely packed Au islands.

An example of a selected area-electron diffraction pattern (SAED) of such an Au@ZnO NW is shown in Figure 2b. The ring-shaped diffraction pattern was attributed to the ultrafine polycrystalline nature of the sputtered Au and indicates a fcc crystal structure. Fcc structures are expected for clusters of more than 200 Au atoms.^21^ The nanoparticles should have a high surface step density with low coordinated Au atoms, acting as reactive adsorption sites for the H_2_S molecules.^21,22^

On the other hand, the discrete spot pattern, and the faint Kikuchi lines, also seen in Figure 2b, represent the monocrystalline ZnO NW with hcp crystal structure. The Kikuchi line pattern appeared due to the thickness of the specimen and indicates the crystalline perfection of the CVD-grown ZnO NW.

**Figure 3:**
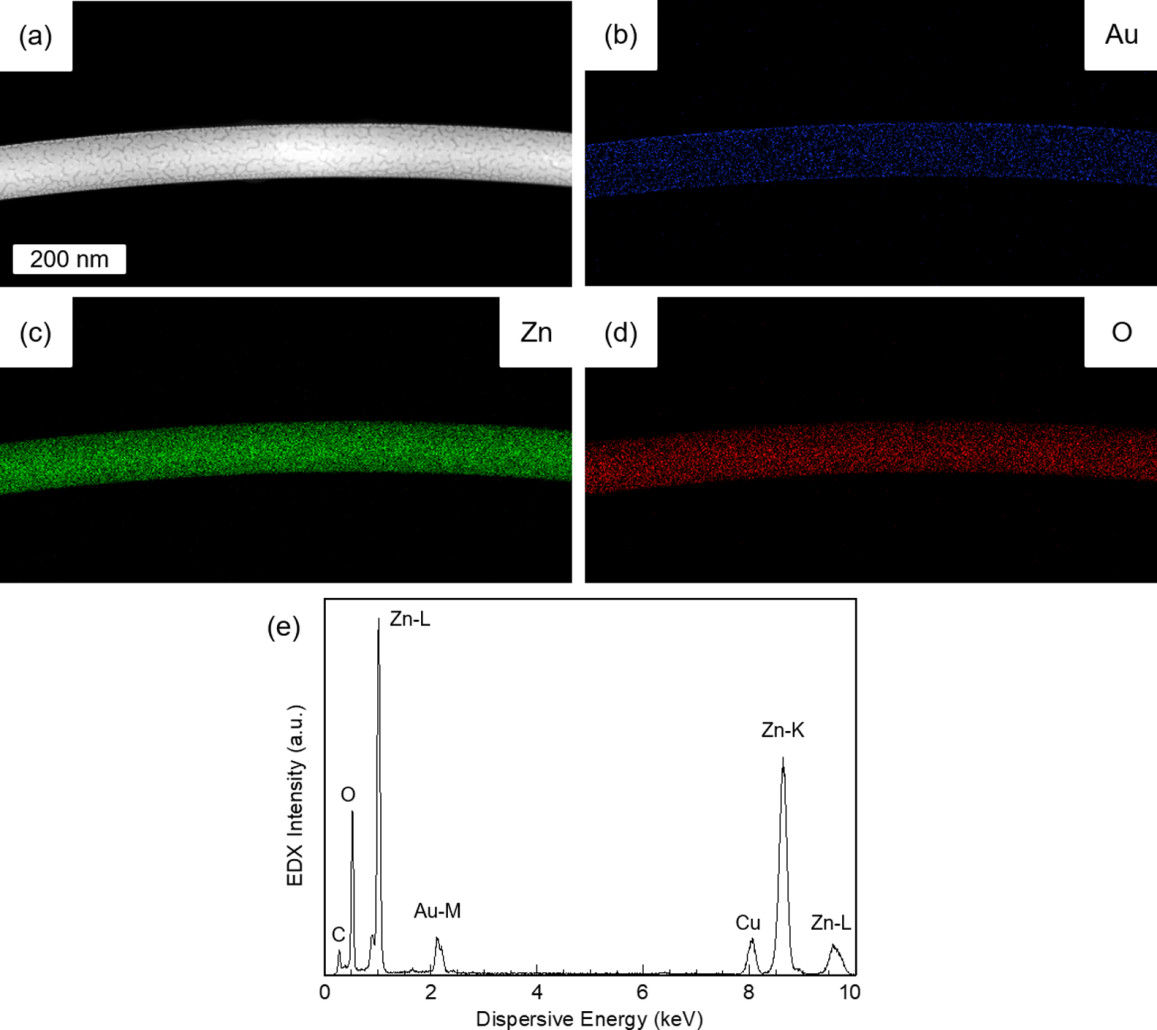
(a) High angle annular darkfield (HAADF) STEM image with corresponding elementary maps (b–d) and integrated EDX spectrum (e) of Au@ZnO NW. The Cu signal originates from stray electrons scattered on the TEM support grid (copper mesh).

Figure 3 shows a high-angle annular dark-field (HAADF) STEM image (Figure 3a) and the corresponding distribution of chemical elements of an Au@ZnO(10–10) NW surface in elementary maps (b–d), and the along the nanowire integrated energy dispersive X-ray spectroscopy (EDX) spectrum (e). In general, the Au nanoparticles appeared to be homogeneously distributed over the entire length of the ZnO NW. The specimen showed no traces of impurities within the detection limit of EDX. The sample contained mainly Zn, O, and Au. The Cu peak originated from the copper TEM grid supporting the ZnO NW specimen.

### 3.2 XPS

XPS analysis was performed to study the impact of H_2_S ambient on the Au surface functionalization as well as to understand its role in the sensing mechanism. Here, both survey and high-resolution XPS spectra for the Au 4f, S 2p, and S 2s regions were recorded. For simplicity, a 5 nm Au functionalization layer was deposited on two silicon wafer pieces (≈ 1 cm^2^). One sample was only exposed to ambient air (Au@Si), the other sample was additionally exposed to 1 ppm of H_2_S(N_2_) for 90 min (Au@Si(H_2_S)). Both samples were kept at room temperature.

**Figure 4:**
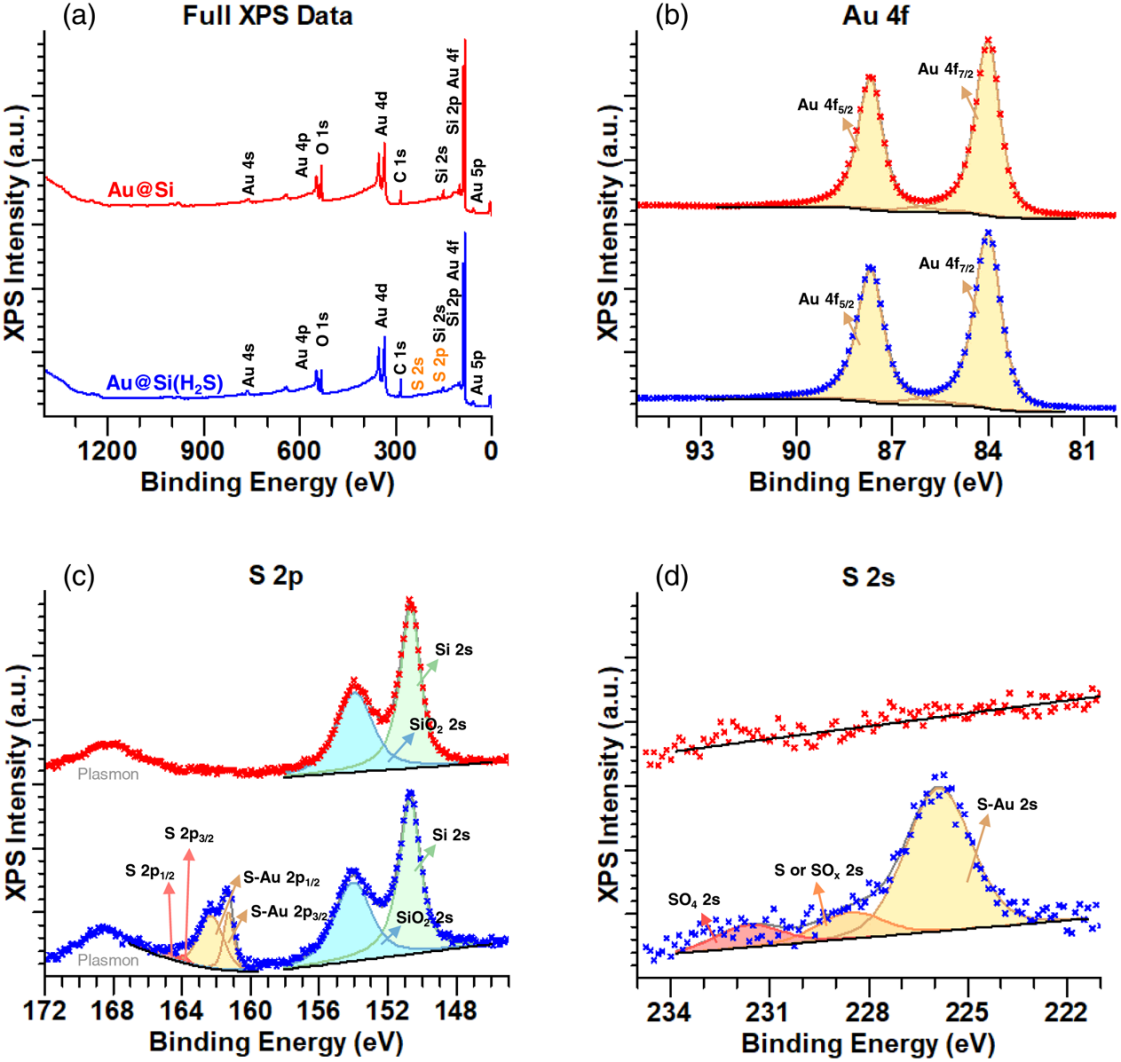
(a) Survey XPS spectra of pristine (red) and Au surface functionalized (blue) samples. (b–d) Comparison of high resolution scans in the Au 4f, S 2p and S 2s region of the same samples.

In the XPS spectra, the positions of the peaks of interest were determined relative to the Au^0^ 4f_7/2_ contribution, which was set to 84.0 eV. Our XPS results are summarized in Figure 4 for both samples.

The survey spectra in Figure 4a showed that the sample surfaces contain carbon (C), oxygen (O), silicon (Si), and gold (Au). Additional evident traces of S were only found for the Au@Si(H_2_S) sample.

Figure 4b shows the high-resolution scans of the Au 4f regions for both the Au@Si sample and the Au@Si(H_2_S) sample. The Au^0^ 4f_7/2_ and the Au^0^ 4f_5/2_ peaks (at 84.0 eV and 87.7 eV) showed comparable peak widths in both scans.

To get information on the impact of exposure to H_2_S, we studied the S 2p and Si 2s regions in high-resolution scans, which is shown in Figure 4c. The Si 2s contributions for pure Si (at 150.7 eV) and silicon oxide (SiO_2_) (at 154.0 eV) were observed for both the Au@Si sample and the Au@Si(H_2_S) sample. Hence, both silicon wafer pieces were covered by an oxidized top layer. The essential difference between both scans occurred in the S 2p peak, which was clearly observed only for the Au@Si(H_2_S) sample. Here, strong S 2p_3/2_ and S 2p_1/2_ contributions at binding energies (BE) of about 161.4 eV and 162.5 eV, respectively, indicate the formation of a sulfide,^23^ namely Au-bonded S, as a direct consequence of the exposure to rather diluted H_2_S. Weaker contributions were observed at 162.7 eV and 163.8 eV, respectively. These contributions could indicate atomically adsorbed S.^23^ However, the low intensity of these contributions did not allow for proper identification. Attributing intensities to further sulfur contributions in the S 2p region appeared to be challenging due to the overlap of S 2p peaks and Si 2s peaks: For example, the prominent contribution at 168 eV was associated with the Si 2s bulk plasmon with a plasmon loss energy of 17 eV.^24^ Sulfate (SO_4_) and sulfite (SO_3_) would also be expected at 168–169 eV^25^ and 166–167 eV,^25^ respectively. The overlap of the Si plasmon contribution with possible SO_4_ or SO_3_ contributions made an identification of the latter in the S 2p and Si 2s region challenging. In contrast, no overlap was expected in the S 2s region, and an identification was more feasible. The high-resolution scans for the S 2s region are displayed in Figure 4d. No significant S 2s contributions were observed for Au@Si. However, for Au@Si(H_2_S) three separate S 2s contributions at 225.9 eV, 228.8 eV, and 232.3 eV were successfully identified. The BE lines for the Au@Si(H_2_S) sample were stronger in intensity and indicated Au-bonded S and Au-bonded SO_4_.^26,27^ The additional third contribution could represent the formation of SO_*x*_. The low intensity of this peak, alongside other contributions located close to each other, made a clear assignment difficult.

In conclusion, as a result of H_2_S exposure, Au-bound S and SO_4_ were clearly observed on Au-functionalized surfaces. Exposing an Au nanoparticle layer to low concentrations of H_2_S at room temperature easily results in surface reactions between Au and S, and a stable attachment of sulfur. This can partially be attributed to the chemical affinity between Au and S,^28^ and to the enhanced surface roughness of our nanoparticle layer with highly reactive low-coordinated Au atoms, as stated in section 3.1. Both effects will facilitate a reaction between our functionalization layer and H_2_S, which leads to the observed XPS contributions. Here, a sulfur adlayer on top of the gold substrate in the form of a strong covalent Au–S bond is expected.^21^

### 3.3 Gas Sensing Properties of Pristine ZnO NWs and Au@ZnO NWs

#### 3.3.1 Irreversible Adsorption of H_2_S

Lee et al.^17^ discussed two simplified adsorption models for any given nanotube sensor array, covering cases of irreversible and reversible adsorption. More importantly, a straightforward analytical test was introduced that allows us to distinguish whether a gas sensor shows reversible or irreversible adsorption of the target gas by analyzing the response transient.

The detection signal toward a target gas of a gas sensor with reversible adsorption dynamics will completely recover and return to its initial base level by simply removing the analyte from the surrounding gas phase. Here, the magnitude of the response correlates with the concentration of the detected target gas, and the sensor is characterized by a detection limit. This detection limit itself is temperature-dependent and defined by an equilibrium coverage, which results from the dynamic equilibrium of ongoing adsorption and desorption processes of the target gas.

On the contrary, the signal magnitude of a sensor with an irreversible adsorption process is not directly related to a target gas concentration, nor does the sensor have a representative detection limit. What appears as a trade-off, enables the detection of extremely small target gas concentrations at prolonged measurement times. As a direct result of missing desorption from surface sites, detection of sub-ppm or sub-ppb concentrations with an irreversible sensor becomes feasible, while reversible sensors would already be equilibrium limited at such low concentrations. Irreversible sensors are examined best by reporting the slope of the measured response.

**Figure 5:**
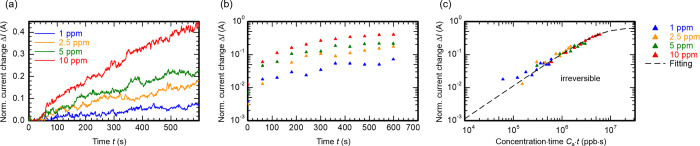
Evaluation of H_2_S adsorption dynamics of our pristine ZnO(10–10) NW surface analogously to the suggested evaluation method of Lee et al.^17^ (a,b) Current change in pristine ZnO NWs at room temperature in response to a set of low H_2_S concentrations. (c) Plotting the current change as a function of *C*_a_·*t* reveals a single model curve, representative for irreversible adsorption dynamic of our gas sensors.

For a correct interpretation of the *PL*-*t* and *I*-*t* data in this work, a detailed understanding of the adsorption dynamics of pristine ZnO NWs and Au@ZnO NWs beforehand is crucial. Hence, we apply the suggested evaluation method of Lee et al. to exemplary gas sensing data of an electrical ZnO NW sensor of our group:

Figure 5a shows the current change of said exemplary electrical ZnO NW sensor exposed to different H_2_S concentrations. To improve the readability, Figure 5b shows the same *I*-*t* data using a semilogarithmic scale and a reduced number of data points. The current change of each concentration is easily distinguishable by its slope or magnitude within the 10 min long detection interval. However, when plotting the current change against the product of target gas concentration *C*_a_ and the ongoing detection time *t*, the different curves in Figure 5a,b appear as a single curve in Figure 5c. According to Lee et al., this behavior is a signature of irreversible adsorption and demonstrates that the interaction of H_2_S with the ZnO(10–10) surface of our sensor is an irreversible process.^17^ Furthermore, findings in our previous work^29^ confirm the irreversible nature of H_2_S detection on ZnO(10–10) surface: Here, we have shown that removing the target gas from the surrounding atmosphere and simply flushing the electrical sensor with N_2_ does not lead to a recovery of the initial signal. Instead, a recovery was only possible by exposing the sensor to O_2_.

Unfortunately, a similar analysis of the current change transient of H_2_S detection via Au@ZnO NWs was not possible, but also not necessary: Based on our previous publications^18,19^ and the XPS data in section 3.2, H_2_S detection on the Au@ZnO(10–10) surface is characterized by the formation of a strong chemical bond between Au and S, which is typical for irreversible chemisorption of H_2_S.

Based on these findings, the optical and electrical H_2_S sensing properties of pristine ZnO NWs and Au@ZnO NWs will be evaluated based on irreversible adsorption dynamics in the following.

#### 3.3.2 Optical and Electrical Gas Sensing

**Figure 6:**
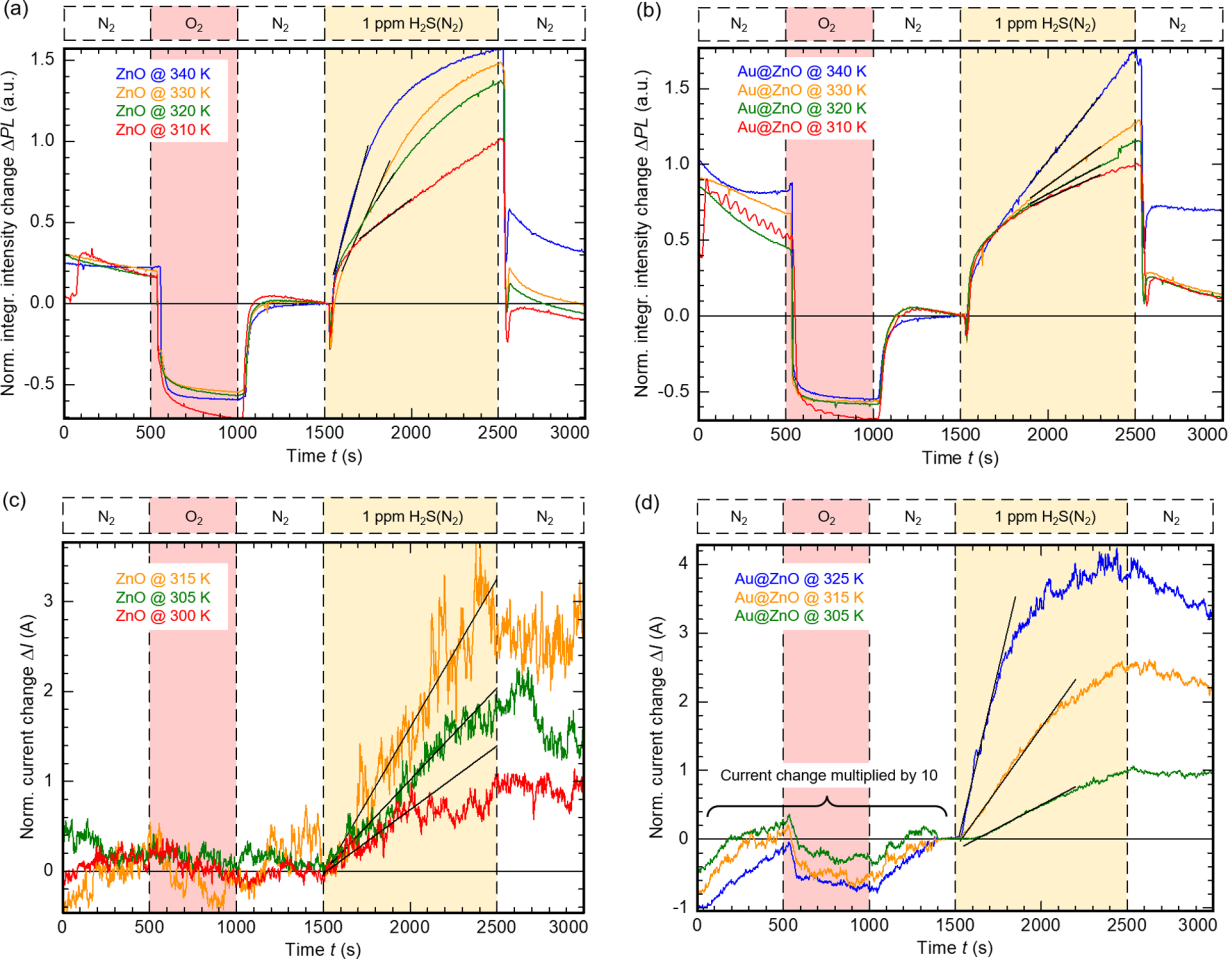
Optical sensing results of (a) pristine ZnO NWs and (b) Au@ZnO NWs recorded at temperatures 310–340 K, and electrical sensing results of (c) pristine ZnO NWs and (d) Au@ZnO NWs recorded at temperatures 300–325 K. The highlighted slopes (black fit curve) are used to estimate the reaction constant *k* and the underlying activation energy *E*_A_.

Optical and electrical gas sensing properties of pristine ZnO NWs and Au@ZnO NWs were investigated by exposing the gas-sensitive samples to the following gas sequence: 500 s of N_2_ (100 %), 500 s of O_2_ (100 %), 500 s of N_2_ (100 %), 1000 s of H_2_S (1 ppm in N_2_), and 500s of N_2_ (100 %). To determine apparent activation energies *E*_A_s of gas adsorption dynamics introduced by Lee et al.,^17^ which would be representative of H_2_S detection at room temperature on either the ZnO(10–10) or the Au@ZnO(10–10) surface, the gas sequence was repeated at different temperatures. The selected temperatures were 310 K, 320 K, 330 K, and 340 K for the optical measurements, and 305 K, 315 K, and 325 K for the electrical measurements, respectively. The resulting *PL*-*t* data are shown in Figure 6a,b, and the *I*-*t* data are shown in Figure 6c,d. The original measurement data were normalized to *PL*_0_ and *I*_0_, as stated in eqs 3 and 4 in section 2.5. *PL*_0_ and *I*_0_ were acquired from the second N_2_ interval. Here, the response of pristine n-type ZnO and Au@ZnO to oxidizing O_2_ appeared as a negative signal and interaction with reducing H_2_S generated a positive signal.

All gas-sensitive samples responded to the gas flushing sequence as expected from literature reports^30^ and our previous works:^18,19^ In an oxidizing gas ambient, the measured signal decreased, while a reducing gas atmosphere increased the initial signal. The response of all samples was clearly detectable even to low trace amounts of H_2_S diluted in N_2_. In comparison to pure N_2_ or pure O_2_, the response toward 1 ppm of H_2_S at any given temperature (for both measurement methods, all samples) was dominating the response signal. While the response toward N_2_ and O_2_ was mostly unaffected by the functionalization for both measurement methods, the response toward H_2_S was clearly enhanced by functionalization with Au.

This enhancement is most pronounced for electrical gas sensing (Figure 6d): Here, an initial response of *R*_norm._ = (3.3 ± 0.1) (Figure 6c) increased to *R*_norm._ = (4.2 ± 0.1) at 325 K (Figure 6d). This is even more noticeable when comparing the non-normalized amplitudes of electrical gas sensing before and after surface functionalization with Au: At 315 K the measured current change Δ*I* for pristine ZnO NWs toward H_2_S was (0.8 ± 0.3) × 10^–8^ A, which increased to (6.9 ± 0.3) × 10^–7^ A for Au@ZnO NWs. Simultaneously, this can be seen when comparing the non-normalized slopes of both electrical gas sensing measurements: At 315 K the measured slope of the H_2_S current change was (8.8 ± 0.1) × 10^–12^ A/s for pristine ZnO NWs, and (1.06 ± 0.01) × 10^–9^ A/s for Au@ZnO NWs. This enhanced signal amplitude leads to a higher signal-to-noise ratio for sensors based on Au@ZnO NWs, which is highly beneficial for an improved limit-of-detection. A detailed analysis and discussion of the impact of surface functionalization of ZnO NWs on the gas detection signal, including data on signal-to-nose, base current change, limit of detection, can be found in our previous studies.^18,19^

For optical gas sensing, the normalized response did not change significantly after Au functionalization: The initial optical response toward 1 ppm of H_2_S gas at 340 K increased from *R*_norm._ = (1.5 ± 1.0) (Figure 6a) to *R*_norm._ = (1.8 ± 0.5) (Figure 6b). Here, the non-normalized slope of the intensity change decreased after the functionalization. This may be explained by the Au nanoparticle layer partially blocking the PL emission of recombining excitons.

Despite applying the same magnetron sputtering parameters for optical and electrical sensing samples, Au functionalization seemed to be more effective for the latter. One reason for a less pronounced increase in the optical response of Au@ZnO NWs may be related to the difference in sample geometry: optical sensor samples were as-grown high-density arrays of upright nanowires, whereas electrical sensor samples consisted of flat laying nanowires distributed on a planar contact structure. As shown in the TEM image in Figure 2a in section 3.1, surface functionalization of ZnO NWs on a planar substrate resulted in a mostly uniform distribution of the noble metal along the nanowire length. For optical gas sensing samples, the Au deposition process is expected to result in a much less homogeneous metal coverage along the nanowire length due to shadowing effects. Hence, for a functionalized optical gas sensing sample, only partial coverage of the ZnO(10–10) surface with Au can be expected. The recorded excitonic NBE emission in PL spectra of Au@ZnO NWs originated more likely from a mixture of pristine and Au functionalized ZnO(10–10) surfaces and thus resulted in a less evident impact of Au surface functionalization on optical H_2_S detection.

Focusing on the temperature series, some striking observations can be made: Optical and electrical measurements were performed in the low-temperature regime, which means that gas sensing was performed at temperatures below 100 °C, and thus significantly below the optimum reactive temperature of ZnO with H_2_S of 200–400 °C. In the investigated temperature range the H_2_S gas sensing signal nonetheless showed pronounced thermal activation: Increasing the sensing temperature by merely 20–30 K improved the magnitude of the measured H_2_S response by a factor of 1.5–3 for both optical and electrical measurements for pristine ZnO NWs and Au@ZnO NWs. This further hints toward mostly irreversible adsorption for all four measured temperature series.^17^

Lee et al. modeled the dynamics of irreversible adsorption based on the following basic surface reaction:
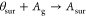
5where *A*_g_ is the analyte molecule in the gas phase, and θ_sur_ and *A*_sur_ represent the concentration of unoccupied and occupied adsorption sites of the analyte on the sensor surface. In our case the analyte is H_2_S and its concentration (particles per volume) in the gas phase is a constant *C*_a_ due to dynamic flushing. This forward reaction is characterized by a reaction rate constant *k*.

Considering, that the total number of adsorption sites *T*_sur_ is conserved:
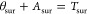
6and the measured response *R*_norm._ is directly proportional to *A*_sur_ (as well as, the maximum possible response *R*_max_ is directly proportional to *T*_sur_), the sensor response is expressed by
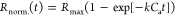
7with *t* as the ongoing measurement time. A detailed derivation of eq 7 can be found in ref (17).

As mentioned in section 3.3.1, the response of irreversible sensors is best determined by evaluating the slope of their response for *t* = 0:^17^
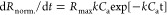
8
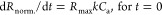
9Here, the slope becomes directly proportional to the reaction rate constant *k*. Figure S3 shows an exemplary gas sensing measurement with a linear fit for the estimation of *k*.

Based on the Arrhenius equation for first-order processes the relation between the reaction rate constant *k* and temperature *T* is
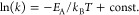
10with *E*_A_ as the activation energy of the temperature-dependent H_2_S interaction with the ZnO NW or Au@ZnO NW surface, and const. = −ln(*R*_max_*C*_a_), which relates to the individual surface size of each sensor.

**Figure 7:**
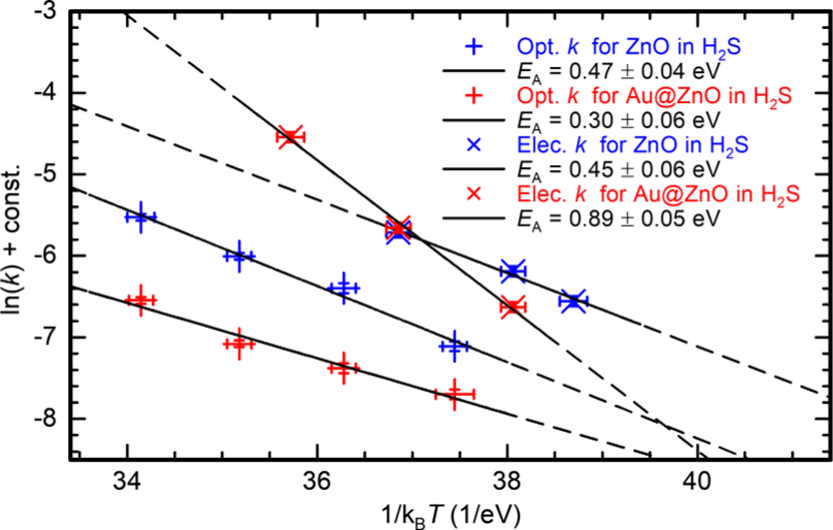
Display and comparison of Arrhenius plot results with derived *E*_A_s for optical (+) and electrical (×) H_2_S gas sensing with pristine ZnO (blue) and Au@ZnO (red) (const. = ln(*R*_max_*C*_a_), and relates to the individual surface size of each sensor). H_2_S detection on pristine ZnO surface is characterized by a low *E*_A_ of ∼0.45 eV, while for electrical H_2_S sensing with functionalized ZnO the *E*_A_ of the crucial gas sensing process is doubled.

Using eqs 9 and 10, the apparent activation energy *E*_A_s for H_2_S adsorption dynamics on pristine ZnO(10–10) surface and on the functionalized Au@ZnO(10–10) surface was estimated from the *PL*-*t* data in Figure 6a,b and the *I*-*t* data in Figure 6c,d. The values of *E*_A_s determined for optical and electrical H_2_S gas sensing are summarized in the Arrhenius plots in Figure 7. From the slope, we derived for optical measurement with pristine ZnO NWs an activation energy *E*_A_ = (0.47 ± 0.04) eV, and for the Au functionalized surface a slightly lower value of *E*_A_ = (0.30 ± 0.06) eV. For the electrical measurement, the equivalent activation energies for H_2_S adsorption were *E*_A_ = (0.45 ± 0.06) eV and *E*_A_ = (0.89 ± 0.05) eV, respectively.

It is evident, that a noticeable difference in *E*_A_s between pristine and functionalized ZnO NWs was observed only for electrical H_2_S gas sensing, but not for optical H_2_S gas sensing. The clearly different slope indicates the possibility of different surface interactions for H_2_S on pristine ZnO and for H_2_S on Au@ZnO. The lack of similar results from the optical data can be explained, by the already mentioned less efficient surface functionalization of ZnO NW growth samples. However, the measurement series of electrical gas sensing via pristine ZnO NWs was much noisier than any other measurement series, as well as it was missing results for the last temperature step of 325 K due to early deterioration of the sensor. This might raise doubts about the legitimacy of the evaluated activation energy of *E*_A_ = (0.45 ± 0.06) eV and the resulting difference in *E*_A_s before and after surface functionalization for electrical gas sensing. Hence, we included data from another exemplary temperature series of electrical H_2_S sensing, which was performed with a different ZnO growth sample, at different temperatures (but still in the low-temperature regime), and with extended flushing times. The comparison, consisting of gas sensing measurement and evaluated *E*_A_s, is shown in Figure S4. For the additional electrical measurement with pristine ZnO NWs, the calculated activation energy was *E*_A_ = (0.47 ± 0.06) eV, which confirms the original result. It can be assumed that in both measurements the same surface interaction for H_2_S dominates. Moreover, this showed that H_2_S detection on ZnO(10–10) surface is characterized by a rather low activation energy for both optical and electrical measurement methods alike, which also points to a generically identical sensing mechanism. Meanwhile, for electrical sensing with Au@ZnO NWs, the activation energy drastically changed after Au functionalization to the point of *E*_A_ being doubled.

Referring to the literature, the calculated *E*_A_s can be attributed to the dissociative chemisorption of H_2_S on either a relaxed ZnO(10–10) surface or a defective Au surface.^31,32^ At room temperature, kinetically fast decomposition upon adsorption of the H_2_S molecule becomes facile on both materials. In our case, due to the low diameter of the ZnO NWs, the ZnO (10–10) surface should be relaxed. Also, the Au nanoparticle layer with its high number of low coordinated Au surface atoms qualifies as a defective Au surface.

Table 1 displays a comparison of our experimental findings and theoretical values for *E*_A_ reported in literature.

**Table 1:** Comparison of Activation Energies *E*_A_ of Dissociative Chemisorption of H_2_S on Pristine ZnO and Au-Related Surface Presented in This Work and Recent Reports in the Literature^a^

material	surface	acquisition type	*E*_A1_	*E*_A2_	*E*_A_	references
ZnO	perfect	theoretical	0	0.53		(31)
	ZnO(10–10)					
ZnO	ZnO(10–10)	theoretical	0	0.51		(33)
				0.55		
Au	defective	theoretical	0.72			(32)
	Au(111)		0.75			
Au	Au(111)	theoretical	0.72	0.68		(34)
ZnO	ZnO(10–10)	experimental optical			0.47 ± 0.04	this work
Au@ZnO	fcc Au on ZnO(10–10)	experimental optical			0.30 ± 0.06	this work
ZnO	ZnO(10–10)	experimental electrical			0.45 ± 0.06	this work
					0.47 ± 0.06	
Au@ZnO	fcc Au on ZnO(10–10)	experimental electrical			0.89 ± 0.05	this work

a *E*_A1_ and *E*_A2_ are the activation energies for the dissociation of the first and second H atom, respectively. For the experimental results of this work, partial and full dissociation cannot be distinguished.

Published density functional theory (DFT) results for dissociative chemisorption of H_2_S on ZnO were reported as *E*_A1_ = 0 eV^31,33^ for reaching the first dissociation state (only one H atom dissociates), and a subsequent activation energy of *E*_A2_ = 0.53 eV^31^ or *E*_A2_ = 0.51–55 eV^33^ for the abstraction of the second H atom. For H_2_S dissociation on Au nanoparticles or Au atom clusters, DFT results established activation energies of *E*_A1_ = 0.72–0.75 eV^32,34^ and *E*_A2_ = 0.68 eV^34^ for partial and full dissociation, respectively. Because the ranges of reported *E*_A_s for partial and full dissociation are quite similar to each other, such dissociation characteristics cannot be distinguished within our experimental results. However, the overall reported values of theoretical *E*_A_s for H_2_S dissociative chemisorption on ZnO and on Au fit to our experimental findings remarkably well.

While the dissociative chemisorption on Au for both dissociation steps may seem less efficient according to the significantly higher reaction barrier, our experimental results still showed an enhanced current change after functionalization. This can be understood by considering the different morphology of our pristine ZnO NW surface and the Au@ZnO NW surface. Compared to the monocrystalline ZnO NW surface (diameter ∼110 nm), the Au surface functionalization with a polycrystalline structure (nanoparticle diameter < 20 nm)^35,36^ provides a much higher surface-to-volume ratio. In addition, TEM results predicted a high surface roughness with highly reactive low coordinated atoms for the Au nanoparticle layer (section 3.1). The resulting abundance of suitable H_2_S adsorption and dissociation sites on the Au@ZnO NW surface may explain the enhancement of the observed signal. In addition, this surface reactivity is unique to sulfur compounds, because of the chemical affinity between Au and S.^28^ Since H_2_S is the sulfur compound in exhaled human breath with the highest concentration,^1^ an Au@ZnO NW offers excellent selectivity by combining the affinity of ZnO toward H_2_S, and the affinity of Au nanoparticles toward S together with the effects of enhanced surface-to-volume ratio. This results in an outstanding and reliable selective detection material for medical H_2_S sensing.

Au can interact with S due to a chemical affinity, but it is unlikely to undergo a reaction with most other species. While this provides a good selectivity for H_2_S sensing, the reversibility of an Au@ZnO-based sensor becomes challenging. Hence, unlike the irreversible H_2_S dissociation on ZnO, which can be reverted by the introduction of O_2_,^29^ the dissociation of H_2_S on Au surface is not fully reversible by O_2_ treatment.^19^ Alongside the investigation of Au and S interaction in high-resolution XPS spectra of the S 2p and S 2s regions, mentioned in section 3.2, we also investigated the O 1s for the Au@Si and the Au@Si(H_2_S) sample. The corresponding spectra are shown in Figure S5. It is noticeable, that the freshly prepared sample showed at least two contributions, while the sample with H_2_S exposure shows only one contribution. The common O 1s contribution at 532.3 eV detected in both samples can be assigned to SiO_2_.^37^ The additional O 1s contribution at 530.4 eV can be interpreted as a metal oxide or oxygen adsorbed on a metal surface, meaning O_(ad)_/Au. It appeared that freshly prepared Au nanoparticles suffer from initial O_2_ contamination. However, a similar interaction between O_2_ and Au did not take place when the nanoparticle layer was already stabilized and H_2_S was introduced. Several reports confirm this observation, as it is well understood that neither molecular nor dissociative chemisorption of O_2_ is possible on the Au surface.^38,39^ This explains the incomplete recovery of an Au@ZnO NW sensor after H_2_S dissociation. Finding a reliable recovery method for Au@ZnO NW sensors is of key importance for future applications.

Based on these findings the underlying gas sensing mechanism of pristine ZnO NWs and Au@ZnO NWs is outlined in section 4.

## 4 Discussion

Considering our experimental results regarding sensing material morphology, surface reactivity, adsorption dynamics, gas sensing ability, and apparent activation energies, we can attempt to outline the H_2_S sensing mechanism of high-temperature CVD grown ZnO NWs with gas sensitive (10–10) facets plus sputtered Au nanoparticle layer with fcc crystal structure supported on ZnO NW surface in the following.

**Figure 8:**
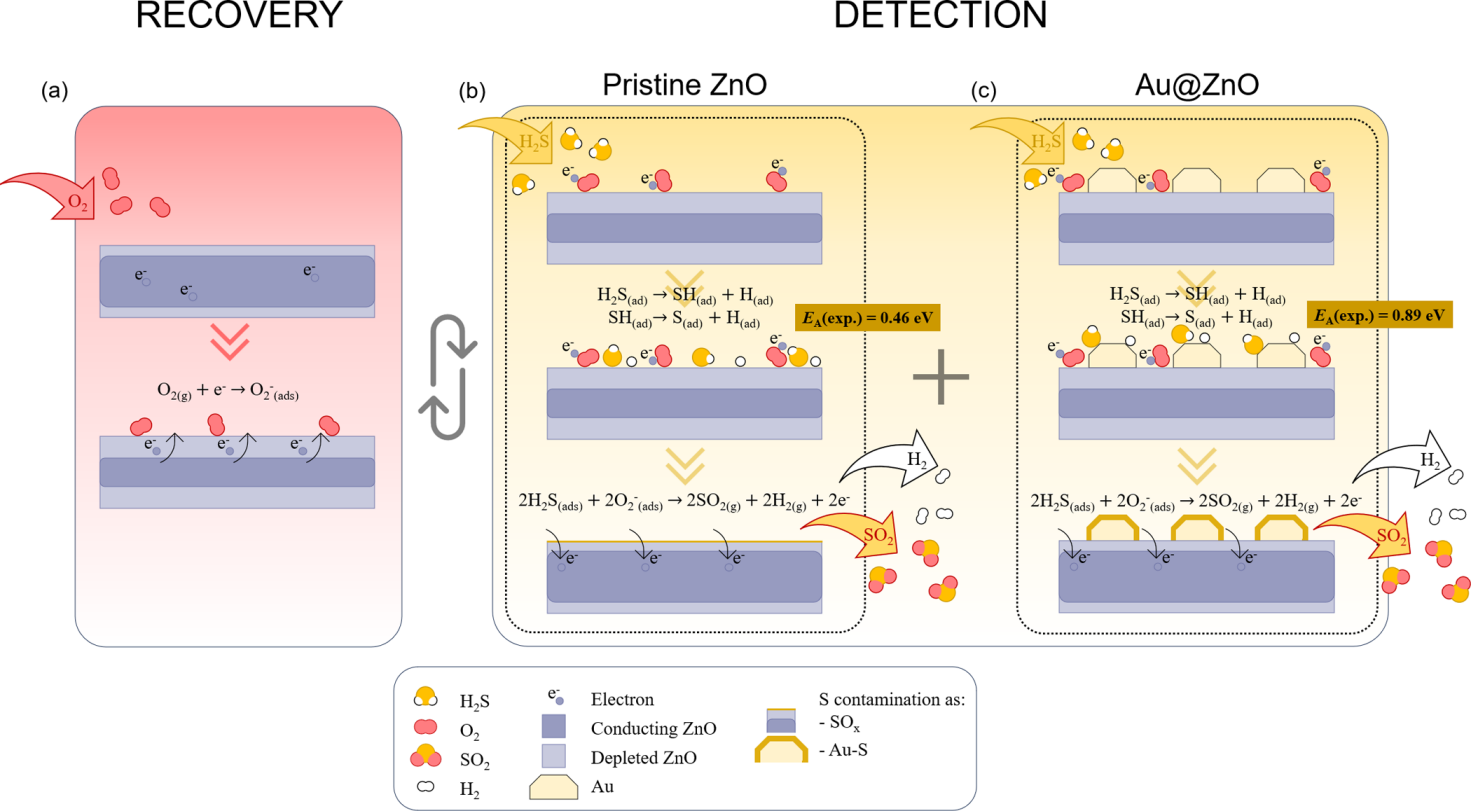
Schematic gas sensing mechanism of ZnO NWs at room temperature. The mechanism consists of (a) sensor recovery in O_2_ rich atmosphere, which is inherent to MOS-based gas sensors, and sensor detection via adsorption and dissociation of the target gas molecule H_2_S on (b) pristine ZnO NW surface or (c) Au nanoparticles (NP) on Au@ZnO surface. Both detection paths are distinguishable by their activation energies *E*_A_s in combination with subsequent S contamination in the form of adsorbed SO_*x*_ species on the ZnO surface or S encapsulation of Au NP by the formation of Au–S bonds.

The proposed sensing mechanism is summarized in Figure 8.

Intrinsic ZnO is an n-type semiconductor due to uncontrolled trace donors and defect states such as oxygen vacancies. Surface states lead to the formation of an electron-depleted ZnO NW surface region, while the ZnO NW core is still electron-rich and conducting.^40^

For optical gas sensing, the recombination of electron–hole pairs and their representative excitonic NBE emission can only originate from the nondepleted ZnO NW core, which is free from high electric fields cracking excitons before recombination in PL experiments. In the case of electrical gas sensing, the measured current originates from the highly conductive ZnO NW core.

Therefore, both the optically measured NBE emission intensity and the electrically measured current from the ZnO NW core are highly dependent on the expansion or reduction of the electron-depleted surface region.

As an initial step of the sensing process, gas sensors based on MOS are commonly exposed to O_2_. Flushing a gas sensor with O_2_ works as a well-defined starting condition and a means of sensor recovery after target gas detection. Once the pristine ZnO(10–10) surface is exposed to O_2_ at room temperature, O_2_ interacts with active adsorption sites and molecularly chemisorbs^41,42^ (Figure 8a). During this adsorption process, additional surface states are formed, which can be filled by electrons from within the conductive ZnO NW core. Consequently, the ZnO NW surface becomes negatively charged, which leads to the formation of an electrical field between the surface and core. This electrical field eventually hinders additional electrons from being trapped by the adsorbed O_2_, and an equilibrium is reached. As a result, the electron-depleted surface region is increased, and the electron-rich ZnO NW core is shrinking.

During optical measurements, this was evident as a decreasing excitonic NBE emission intensity, or as a decreasing current during electrical measurement, respectively.

For the pristine ZnO(10–10) surface, this adsorption process is summarized in the reaction:^40^
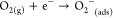
11

Because of Au not interacting with O_2_, as addressed in section 3.3.2, a similar interaction is expected for Au@ZnO NWs: O_2_ will interact and adsorb on the pristine ZnO(10–10) surface only, which would be in-between the Au islands. In conclusion, the starting conditions for H_2_S sensing with pristine ZnO and Au@ZnO are comparable (Figure 8b,c).

During the target gas detection, pristine ZnO NWs and Au@ZnO NWs are exposed to the reducing target gas H_2_S. Upon interaction with the ZnO(10–10) surface, H_2_S molecules undergo dissociative chemisorption at a relatively low activation energy of *E*_A_= 0.46 eV (Figure 8b). On the Au@ZnO(10–10) surface, H_2_S molecules will also chemisorb and dissociate on the more abundant Au nanoparticles requiring a higher activation energy of *E*_A_= 0.89 eV (Figure 8c). For both scenarios, the H_2_S dissociation can be expressed as^33,34^
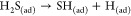
12
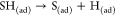
13

which was also evident in our XPS results.

Although, the H_2_S dissociation on Au has a higher *E*_A_ than H_2_S chemisorbed dissociation on ZnO, the Au nanoparticle layer has a higher surface-to-volume ratio than the ZnO NWs. A large abundance of low coordinated surface atoms, as seen via TEM, and easily accessible dissociation sites enhance the number of available and dissociated H_2_S on the sensor surface.

On the pristine ZnO surface, the adsorption energy of O_2_ is very low,^43^ and the chemisorbed molecule stays mobile. Thus, it can easily interact and react with the dissociated H_2_S on pristine ZnO and form SO_2_, H_2_ (or H_2_O):^44^
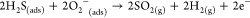
14

Both, SO_2_ and H_2_, are expected to immediately desorb from the ZnO surface, and the previously O_2_-trapped electrons are released back into the ZnO NW core. The width of the electron-depleted surface region then decreases, and the electron-rich core increases in diameter. Consequently, the excitonic NBE emission, as well as the NW current rises.

For the Au functionalized surface, a similar interaction is expected. Since adsorbed O_2_ on ZnO is mobile, it can interact with Au-adsorbed H_2_S at the interface region between the Au nanoparticle layer and the pristine ZnO surface. The reaction in eq 14 will take place. However, S will also react with the entire surface of the Au nanoparticle layer and form strong covalent Au–S bonds. Because O_2_ does not adsorb on Au itself, a complete recovery of Au@ZnO following Figure 8a is not possible. As an inevitable consequence, sulfur contamination takes place. This was investigated in our previous studies,^18,19^ and was now successfully identified via XPS and *E*_a_ evaluation, showing that upon continuous exposure to H_2_S the Au nanoparticle layer will eventually be completely encapsulated by sulfur, and block any further interaction between the target gas and the surface functionalization. Interaction will only be possible with the pristine ZnO NW surface. As a result, Au@ZnO NW sensors show signs of fast deterioration, meaning a fast signal loss toward the target gas H_2_S after just a few H_2_S flushing intervals.

For the pristine ZnO NWs such S contamination is only possible in the form of various SO_*x*_ species which do not fully desorb^44^ or the formation of ZnS.^45^ However, these contamination options are not severe for ZnO NW sensors at operating temperatures below 100 °C.

## 5 Conclusions

Two nanomaterial systems, pristine ZnO NWs and Au decorated Au@ZnO NWs, were successfully prepared and investigated on their H_2_S sensing abilities. The dominating reaction pathways occurring during target gas detection were unveiled by XPS measurements and *E*_A_ estimation.

ZnO NWs with their gas-sensitive (10–10)-surfaces were grown by high-temperature CVD. Au surface functionalization was achieved by the deposition of a 3 nm thick nanoparticle layer via magnetron sputtering at room temperature.

Investigation of material morphology was conducted by SEM and TEM measurements. ZnO NWs had a monocrystalline hcp structure. Au surface functionalization consisted of polycrystalline grains with fcc structure and a high number of low coordinated atoms, acting as suitable H_2_S adsorption sites.

Sensing properties were investigated by photoluminescence intensity-over-time measurements and current-over-time measurements, which led to similar results for pristine ZnO NWs. This pointed to similar sensing kinetics for optical and electrical H_2_S gas sensing at low temperatures. It was further shown, that H_2_S gas sensing is a thermally activated process on ZnO(10–10 and on Au@ZnO(10–10) surface.

Evaluation of the response transient of ZnO NWs and XPS measurements of the Au functionalization confirmed irreversible adsorption dynamics on the surface of both material systems. Especially for Au functionalized ZnO NWs, material contamination with Au-bound S is shown to occur.

Arrhenius evaluation of the temperature-dependent response allowed us to experimentally conceive activation energies for H_2_S adsorption on ZnO NWs (*E*_A_ = (0.45 ± 0.06) eV) and on Au@ZnO NWs (*E*_A_ = (0.89 ± 0.05) eV) for temperatures between RT and 100 °C. By comparison to literature data, these activation energies hint toward irreversible dissociative chemisorption of the H_2_S molecule on both surfaces, which would be in good agreement with the concluded irreversible adsorption dynamics.

Overall, this work significantly contributes to a complete picture of the sensitive and selective H_2_S sensing mechanism of pristine ZnO NWs and Au@ZnO NWs. We successfully link experimentally identified surface interactions (XPS) and absorption dynamics (*E*_A_) to a theoretically predicted sensing mechanism by using electrical and optical sensing. Our findings especially explain the impact of Au-functionalization on H_2_S sensing, including its morphology, and emphasizes the superior response and selectivity of Au-functionalized ZnO NWs for medical H_2_S detection in human breath.

## Supporting Information Available

Additional exemplary optical and electrical gas sensing measurements and methods, including experimental details regarding irreversible H_2_S adsorption (PDF)
