# Supplementary material for: Mechanism of Optical and Electrical H2S Gas Sensing of Pristine and Surface Functionalized ZnO Nanowires
Source: ACS Omega. 2024 Dec 12;9(51):50188–200. doi: 10.1021/acsomega.4c04412 (PMC11683626; doi:10.1021/acsomega.4c04412)
Supplement: Supplementary file 1 — ao4c04412_si_001.pdf [file ao4c04412_si_001.pdf]

Mechanism of optical and electrical H_2S gas sensing of pristine and surface functionalized ZnO nanowires

Supporting Information

Angelika Kaiser [a, b, *], Tanja Mauritz [a, b], Joachim Bansmann [c], Johannes Biskupek [d], Ulrich Herr [a], and Klaus Thonke [b]

[a] *Institute of Functional Nanosystems, University Ulm, 89081 Ulm, Germany*

[b] *Semiconductor Physics Group, University Ulm, 89081 Ulm, Germany*

[c] *Institute for Surface Science and Catalysis, University Ulm, 89081 Ulm, Germany*

[d] *Electron Microscopy Group of Materials Science, University Ulm, 89081 Ulm, Germany*

* *corresponding author email: angelika.kaiser@uni-ulm.de*

Supplementary: Figures and Tables

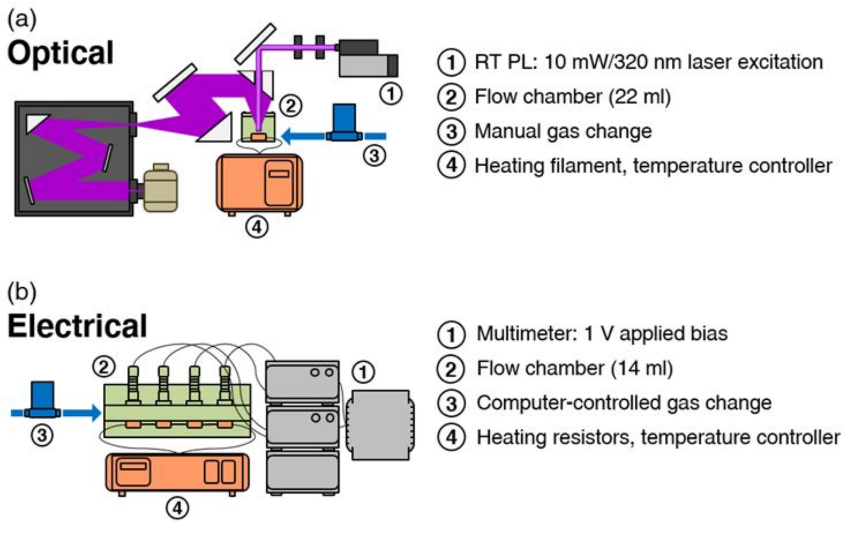

Figure S1. Schematic depiction of (a) optical and (b) electrical measurement setup.

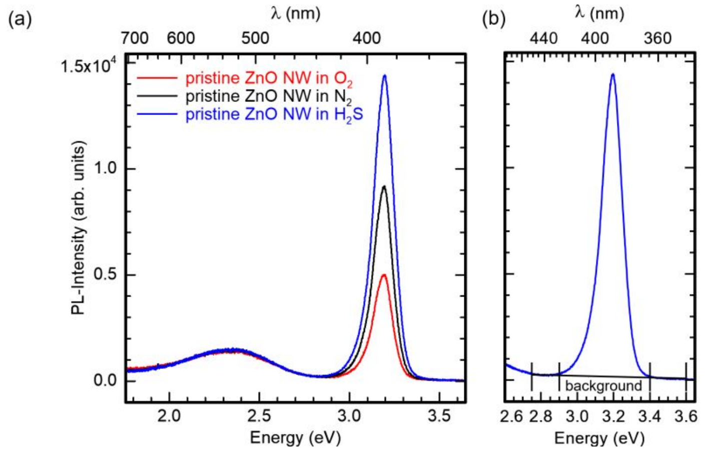

Figure S2. (a) Exemplary room temperature PL spectra of pristine ZnO NWs in different gas atmospheres. Spectra are not normalized. The two main contributions are the near-band-edge emission (NBE) at 3.21 eV, and the broad green band emission centered at ~2.45 eV, which does not change with ambient conditions. The NBE intensity is affected greatly by the surrounding atmosphere, hence the optical gas sensing signal was interpreted as (b) the integrated NBE intensity.

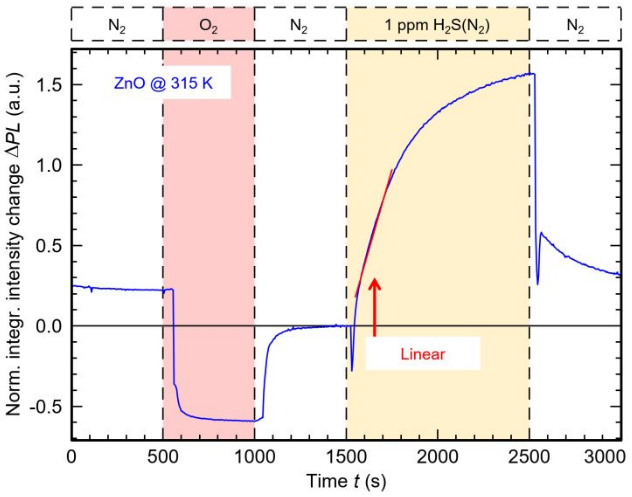

Figure S3. Exemplary gas sensing measurement with its characteristic H_2S response. In addition, a linear fit for the estimation of the temperature-dependent reaction constant *k* is displayed.

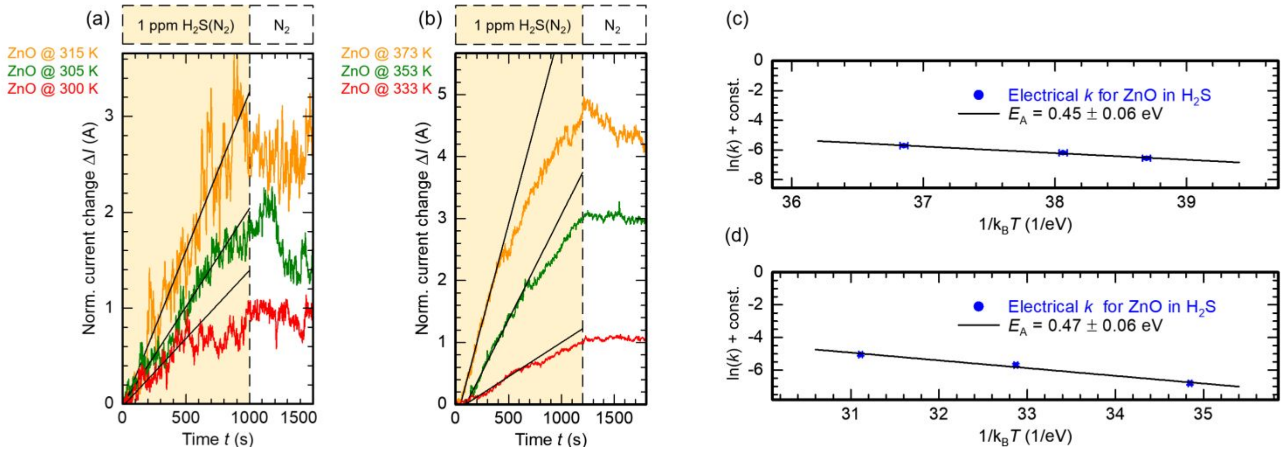

Figure S4. Comparison of (a) electrical H_2S sensing data from Figure 6(c) with (b) additional exemplary temperature series of electrical H_2S sensing, which was performed with a different set of pristine ZnO NW. (c) E_A from Figure 7(d) E_A for the additional exemplary measurement (const. = $\ln(R_{max}C_a)$, and relates to the individual surface size of each sensor). The additional measurement confirms the result.

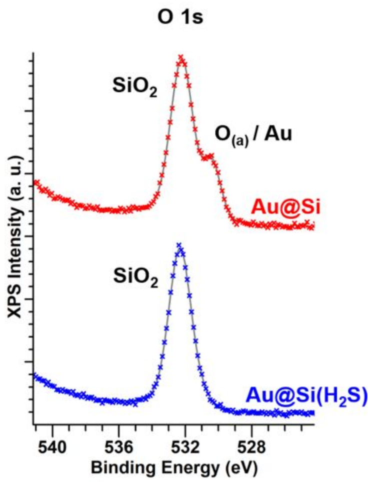

Figure S5. Comparison of high-resolution XPS spectra in the O 1s region of pristine and Au surface functionalized samples.

Author Information

Corresponding Author

Angelika Kaiser – *Institute of Functional Nanosystems, University Ulm, 89081 Ulm, Germany; Semiconductor Physics Group, University Ulm, 89081 Ulm, Germany;*

Email: angelika.kaiser@uni-ulm.de

Authors

Tanja Mauritz – *Institute of Functional Nanosystems, University Ulm, 89081 Ulm, Germany; Semiconductor Physics Group, University Ulm, 89081 Ulm, Germany;*

Joachim Bansmann – *Institute for Surface Science and Catalysis, University Ulm, 89081 Ulm, Germany;*

Johannes Biskupek – *Electron Microscopy Group of Materials Science, University Ulm, 89081 Ulm, Germany;*

Ulrich Herr – *Institute of Functional Nanosystems, University Ulm, 89081 Ulm, Germany;*

Klaus Thonke – *Semiconductor Physics Group, University Ulm, 89081 Ulm, Germany;*
